# Role and mechanism of organic cation transporter 3 in oxaliplatin treatment of colon cancer *in vitro* and *in vivo*

**DOI:** 10.3892/or.2019.7267

**Published:** 2019-08-07

**Authors:** Juan Gu, Ling Wang, Tingting Li, Shiwei Tang, Yuhe Wang, Wei Zhang, Xuehua Jiang

**Affiliations:** 1 Department of Pharmacy, Affiliated Hospital of Zunyi Medical University, Zunyi, Guizhou 563003, P.R. China; 2 Department of Clinical Pharmacy, West China School of Pharmacy, Sichuan University, Chengdu, Sichuan 610041, P.R. China; 3 Department of Pharmacy, People's Hospital of Xishuangbanna Dai Autonomous Prefecture, Jinghong, Yunnan 666100, P.R. China; 4 Department of Critical Care Medicine, Affiliated Hospital of Zunyi Medical University, Zunyi, Guizhou 563003, P.R. China

**Keywords:** OCT3, SLC22A3, colon cancer, oxaliplatin, decitabine

## Abstract

Oxaliplatin (OXA) is routinely used as the first-line treatment for colorectal cancer (CRC). The addition of OXA to chemotherapy has significantly improved the prognosis of patients with CRC; however, some cases are resistant to OXA. The present study explored the influence of organic cation transporter 3 (OCT3) expression on the effects of OXA on CRC cell viability, and investigated the direct effects of OCT3 on viability, invasion and migration of CRC cells using MTT assay, wound healing assay, reverse transcription-quantitative polymerase chain reaction, inductively coupled plasma mass spectrometry and lentiviral interference. The results demonstrated that OXA cellular concentration and OXA-induced cytotoxicity were significantly increased in response to high expression of OCT3, whereas OCT3 knockdown directly increased the invasion and migration of colon cancer cells. Furthermore, upregulation of OCT3 expression in colon cancer xenografts via treatment with the DNA methyltransferase inhibitor decitabine increased cellular OXA concentration and improved the curative effect of OXA. These results collectively indicated that OCT3 may enhance the effects of OXA in CRC cells and may directly inhibit their invasion and migration. Therefore, OCT3 may be a therapeutic target in patients with CRC.

## Introduction

Oxaliplatin (OXA) is a widely used third-generation platinum anticancer drug that is becoming the standard-of-care in the management of colorectal cancer (CRC). Conversely, other platinum-based drugs lack anticancer efficacy in CRC (1). However, many patients do not benefit from OXA treatment (2). Buss *et al* (3) indicated that a reduction in the influx rate of OXA has been observed in resistant CRC cell lines; however, no difference in efflux rate exists between sensitive and resistant CRC cell lines. In addition, Oguri *et al* (4) demonstrated that the intracellular accumulation of OXA in PC-14 cells resistant to OXA is reduced compared with in parental cells; however, no difference has been reported in the expression of efflux transporters SLC47A1 and SLC47A2. Organic cation transporter 3 (OCT3), which belongs to the solute carrier 22 member family and is encoded by solute carrier family 22 member 3 (*SLC22A3*), is critical for drug transportation and cellular detoxification (5,6). The expression of OCT3 in CRC cell lines is higher than that of other organic cation transporters, and the higher the expression of OCT3 in CRC cell lines, the higher the concentration of OXA in the cells (7), suggesting that OCT3 may have a role in the uptake of OXA, although the results are contradictory (8). Whether the expression and regulation of OCT3 influences the effect of OXA on CRC and the possible mechanisms remain to be investigated. A recent study suggested that *SLC22A3* may be a tumor suppressor gene (9–12). Fu *et al* demonstrated that *SLC22A3* suppresses esophageal squamous cell carcinoma metastasis by inhibiting epithelial-mesenchymal transition (EMT) (12). Guo *et al* reported that OXA-resistant HCT116 CRC cells exhibit an EMT phenotype characterized by upregulated expression of matrix metalloproteinase (MMP)2 and MMP9, and downregulated E-cadherin expression (13). Further investigation is required as to whether OCT3 can increase the effects of OXA on CRC by inhibiting the EMT of CRC cells. CRC is the third most common cancer worldwide (14). At diagnosis, ~20% of patients with CRC have distant metastases, with 30–40% having developed vascular and lymph node metastasis (15). Therefore, the discovery of targets that can affect the malignant behavior of CRC is of great significance in developing novel preventative and treatment strategies.

Aberrant DNA methylation is a potential mechanism underlying the development of CRC and reflects the chemosensitivity of patients receiving postoperative adjuvant chemotherapy (16–19). DNA methylation is an epigenetic process that can lead to silencing of gene expression and can be reversed by DNA-demethylating agents. Analysis of *SLC22A3* using MethPrimer (20) revealed that CpG islands exist in its promoter region, suggesting that regulation of this gene by methylation may have a role in its expression. The DNA methyltransferase inhibitor decitabine (DAC) is a Food and Drug Administration-approved drug used clinically to treat acute myelodysplastic leukemia, and has been reported to inhibit cell invasiveness and proliferation of CRC lines (21).

The present study aimed to explore the effect and possible mechanisms of OCT3 in OXA treatment of CRC *in vitro* and *in vivo*. Specifically, OCT3 expression was induced in HCT116 cells with low OCT3 expression using DAC, and *SLC22A3* expression was knocked down in HT29 cells with high OCT3 expression via stable lentiviral interference, in order to investigate the effects of OCT3 expression on OXA transport and CRC cell viability. The direct effect of OCT3 on malignant biological behaviors, such as migration and invasion of CRC cells, was investigated using a wound healing assay and reverse transcription-quantitative polymerase chain reaction (RT-qPCR). In addition, the curative effect of DAC combined with OXA in a nude mice xenograft model of CRC was explored and the association between OCT3 expression and OXA concentration was investigated. The concentration of OXA was detected by inductively coupled plasma mass spectrometry (ICP-MS).

## Materials and methods

### 

#### Materials

OXA was purchased from Dalian Meilun Biotechnology Co., Ltd. DAC (5-Aza-2′-deoxycytidine) was purchased from Selleck Chemicals and MTT was purchased from Sigma-Aldrich (Merck KGaA). Solutions of OXA (7 mmol/l) and DAC (40 mmol/l) were freshly prepared in DMEM cell culture media (Gibco; Thermo Fisher Scientific, Inc.).

#### Cell culture

CRC cell lines HCT116, HT29, SW620 and 293T were obtained from the Shanghai Institute of Cell Biology, Chinese Academy of Sciences. HCT116, HT29 and 293T cells were cultured in high glucose DMEM (Gibco; Thermo Fisher Scientific, Inc.), and SW620 cells were cultured in Leibovitz's L-15 (Gibco; Thermo Fisher Scientific, Inc.). Cells were cultured in medium supplemented with 10% fetal bovine serum (FBS; Gibco; Thermo Fisher Scientific, Inc.), 100 U/ml penicillin and 100 µg/ml streptomycin (Gibco; Thermo Fisher Scientific, Inc.). HCT116, HT29 and 293T cell lines were grown at 37°C in a humidified atmosphere containing 5% CO_2_/95% air, whereas SW620 cells were grown at 37°C in a humidified atmosphere containing 100% air. HT29 STR profiles matched the standards recommended for HT29 cell line authentication, with an EV value of 94%.

#### Construction of stable transfected cell lines

A lentiviral vector encoding *SLC22A3* short hairpin RNA (shRNA) was designed and synthesized by Shanghai Yile Biotechnology Company, Ltd. The shRNA negative control sequence, which had no significant homology to human gene sequences, was 5′-CAACAAGATGAAGAGCACCAA-3′. The shRNA sequence targeting *SLC22A3* (sh-OCT3) was 5′-GAGGAAATGCACACTTATTCT-3′ for HCT116 cells and 5′-GAATTGTACCCAACAACATTA-3′ for HT29 cells. These shRNA fragments were cloned into pLv-shRNA-GP vector-Puro (Shanghai Yile Biotechnology Company, Ltd.) to construct lentiviral vectors. A total of 3×10^6^ 293T cells were inoculated in a 6 cm culture dish 1 day prior to transfection. The cells were cultured overnight in a 37°C and 5% CO_2_ to ensure 80–90% cell confluence at the time of transfection. Subsequently, the lentiviral vector (0.6 µg/ml) and auxiliary plasmids (0.6 µg/ml; Gag-Pol:Rev:VSVG ratio of 5:2:3; Shanghai Yile Biotechnology Company, Ltd.) were transfected into 293T cells using Lipofectamine^®^ 3000 (Invitrogen; Thermo Fisher Scientific, Inc.) according to the manufacturer's protocol at a ratio of 1:1 to produce the lentivirus. 293T cells were incubated at 37°C and 5% CO_2_ for 48 h. The supernatant was collected 48 h post-transfection, centrifuged at 4°C and 1,500 × g for 10 min, and filtered through a 0.45-µm microporous membrane to remove cell debris, and temporarily stored at 4°C. A total of 4×10^5^ human CRC cell lines (HCT116 and HT29) were inoculated in a 12-well plate and infected with the viral suspension (100 µl crude viral liquid per well) alongside 6 µg/ml polybrene [Yeasen Biotechnology (Shanghai) Co., Ltd.]; the optimal lentivirus volume was determined through preliminary experiments. A total of 72 h post-infection, the positive stably transduced cell lines were screened using puromycin (Thermo Fisher Scientific, Inc.) at 1 µg/ml for HCT116 cells and 0.5 µg/ml for HT29 cells. RT-qPCR was employed to detect interference efficiency.

#### Cell treatment

For RT-qPCR analysis, a total of 2×10^5^ cells were seeded into 6-well plates, 6 h after which, DAC was added at different density (0.6, 1.25, 2.5, 5 and 50 µM for HCT116 cells; 0.63, 1.25, 2.5, 5 and 50 µM for SW620 cells; 2.5, 5, 20, 50 and 100 µM for HT29 cells) for 72 h. In addition, cells of different concentrations were inoculated into 6-well plates, 2.5 µM DAC was added to HCT116 cells for 24 h (6×10^5^ cells per well), 48 h (4×10^5^ cells per well) and 72 h (2×10^5^ cells per well), and to SW620 and HT29 cells for 48 h (4×10^5^ cells per well), 72 h (2×10^5^ cells per well) and 96 h (1×10^5^ cells per well). Cells were incubated at 37°C and 5% CO_2_.

#### RT-qPCR analysis

Total RNA was isolated from cultured cells (80% confluent) using TRIzol^®^ reagent (Invitrogen; Thermo Fisher Scientific, Inc.) according to the manufacturer's protocol. First-strand cDNA was synthesized using a reverse transcriptase kit (cat. no. TAKARA047A; Takara Biotechnology Co., Ltd.) according to the manufacturer's instructions. Subsequently, qPCR was conducted according to the manufacturer's protocols under the following conditions: Denaturation for 5 min at 95°C, followed by 40 cycles of 15 sec at 95°C, 20 sec at 60°C and 30 sec at 72 °C, and a final extension step of 5 min at 72°C on a Linegene 9620 real-time PCR system (Hangzhou Bioer Technology Co., Ltd.) using SYBR-Green master mix [Yeasen Biotechnology (Shanghai) Co., Ltd.]. Gene-specific primers were synthesized by the Beijing Genomics Institute and are listed in Table I. All samples were normalized against *ACTB* expression. Relative gene expression was analyzed using the 2^−ΔΔCq^ method (22).

#### Western blotting

Cells were lysed in ice-cold RIPA lysis buffer (Beyotime Institute of Biotechnology) containing PMSF, and protein concentrations were detected using a bicinchoninic acid protein assay kit (Beyotime Institute of Biotechnology). A total of 40 µg protein was separated by 10% SDS-PAGE and transferred onto polyvinylidene fluoride membranes (EMD Millipore), which were blocked by soaking in 5% non-fat milk for 1.5 h at 37°C. Subsequently, the membranes were incubated with polyclonal rabbit anti-human OCT3 antibody (1:500; cat. no. OM291394; OminimAbs) or polyclonal rabbit anti-human GAPDH antibody (1:1,000; cat. no. WL01114; Wanleibio Co., Ltd.) overnight at 4°C. After washing with 1X TBS- 0.1% Tween three times (10 min/wash), the membranes were incubated with goat anti-rabbit immunoglobulin G/horseradish peroxidase (1:10,000; cat. no. bs-0295G-HRP; BIOSS) for 1 h at 37°C. Finally, the bands were visualized using an enhanced chemiluminescence kit (EMD Millipore). Signals were semi-quantified by ImageJ software (version 1.8.0; National Institutes of Health) and normalized to GAPDH.

#### Cell viability assay

The cytotoxicity of compounds was examined using the MTT assay (Invitrogen; Thermo Fisher Scientific, Inc.). Cells were seeded into 96-well plates at ~5,000 cells per well, incubated overnight, and treated with 2.5 µM DAC for the following durations: HCT116 for 24, 48 and 72 h; SW620 cells for 48 and 72 h; and HT29 cells for 72 h. OXA was added at different concentrations (0.625, 1.25, 2.5, 10, 20, 50, 200, 800, 3,200 and 6,400 µM for HCT116 cells; 1.25, 2.5, 10, 50, 75, 100, 200, 600, 1,200 and 2,400 µM for SW620 cells; 0.625, 2.5, 5, 10, 25, 75, 150, 300, 350, 400 and 650 µM for HT29 cells) for a further 24 h, and 10 µl MTT reagent was then added to each well and incubated at 37°C for 4 h. After gently removing the culture medium from the 96-well plates, 100 µl dimethyl sulfoxide was added to the 96-well plates, and the crystals were dissolved in a shaker at low speed at 37°C for 10 min. An automatic microplate spectrophotometer (Multiskan MK3; Thermo Fisher Scientific, Inc.) was used to measure absorbance at 490 nm. All experiments were repeated at least three times.

#### Cell migration assay

The bottoms of 9.5 cm^2^ dishes were marked with a pen and ruler prior to cell seeding. Subsequently, control, negative control and sh-OCT3 HT29 cells (1.6×10^5^ cells) were seeded into 9.5 cm^2^ dishes in 1% serum-containing medium, and allowed to reach 90–100% confluence overnight, forming a monolayer. Subsequently, the cells were scratched with a 10-µl pipette tip perpendicularly to the surface to cross the marker lines, forming fixed detection points. Cells were washed with PBS twice followed by the addition of fresh media with 1% FBS. Images were captured at 0, 24, 36 and 48 h using an inverted light microscope.

#### Methylation-specific PCR (MSP) assays

The promoter region of *SLC22A3* was searched for in Genecopoeia (www.genecopoeia.com). MSP primers, as detailed in Table II, were designed using MethPrimer (The Li Lab, www.urogene.org/methprimer) for the promoter region of *SLC22A3*. Genomic DNA extraction and purification was performed using the Genomic DNA Small Purification kit (Wanleibio Co., Ltd.) according to the manufacturer's protocol. DNA bisulfite transformation, purification, and detection of DNA concentration and purity were conducted using a DNA bisulfite transformation kit (centrifugal column type) [cat. no. DP215; Tiangen Biotech (Beijing) Co., Ltd.] according to the manufacturer's protocol. Subsequently, MSP was performed using a MSP kit [cat. no. EM101; Tiangen Biotech (Beijing) Co., Ltd.], according to manufacturer's protocol. After the reaction, 10 µl product was obtained for agarose gel electrophoresis (12% agarose) and the products of gel electrophoresis were retrieved according to E.Z.N.A.^®^ Gel Extraction kit (cat. no. D2500-01; Omega Bio-Tek, Inc.). The PCR product was linked to the pClone007 vector using the pClone007 vector kit (cat. no. TSV-007; Beijing TsingKe Biotech Co., Ltd.) and identified through sequencing by TSINGKE Biological Technology.

#### Xenograft tumor formation assay in nude mice

**Figure 1.**
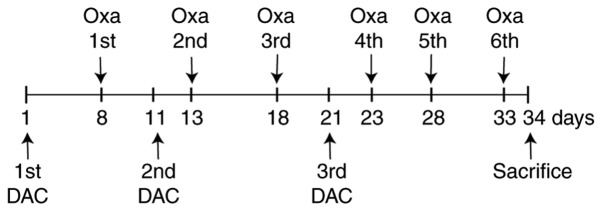
Experimental procedure for animal treatment. A total of 32 mice were divided into four groups (n=8/group): DAC group, OXA group, combination group and control group. DAC (2.5 mg/kg) was intraperitoneally administered into mice in the DAC and combination groups every 3 h on days 1, 11 and 21 for three times a day. OXA (10 mg/kg) was intraperitoneally administered once into mice in the OXA and combination groups on days 8, 13, 18, 23, 28 and 33 Mice in the control group were intraperitoneally administered an equal volume of solvent (5% glucose). DAC, decitabine; OXA, oxaliplatin.

Animal experiments were approved by the Ethical Committee for Animal Research of Sichuan University. HCT116 cells (5×10^6^ cells; 100 µl) were subcutaneously inoculated into the upper right flank of 6-week-old male nude mice (total n=40; weight, ~15 g). BALB/c-nu/nu mice were purchased from Chengdu Dossy Experimental Animals Co., Ltd. The mice were housed under a 12-h light/dark cycle at 25°C with 50–60% humidity and free access to food and water. The length and width of tumors were measured using calipers and the tumor volume was calculated as follows: [(lengthxwidth^2^)/2] twice per week. The relative tumor volume (RTV) was calculated as follows: RTV=V_t_/V_0_. Where V_0_ is the tumor volume at grouping and Vt is the tumor volume at each measurement. Approximately 10 days following inoculation, nude mice with tumor volumes of 100–200 mm^3^ were selected and divided into groups as followings: Control group, DAC group, OXA group and combination group. DAC (2.5 mg/kg) was intraperitoneally administered into mice in the DAC and combination groups every 3 h on days 1, 11 and 21, three times a day. OXA (10 mg/kg) was intraperitoneally administered once into mice in the OXA and combination groups on days 8, 13, 18, 23, 28 and 33. Mice in the control group were intraperitoneally administered an equal volume of solvent (5% glucose). The experimental procedure is shown in Fig. 1. On day 34 mice were sacrificed by cervical dislocation and tumors were collected for the measurement of tumor weights, detection of *SLC22A3* mRNA and protein expression by qPCR and western blotting, respectively, and measurement of OXA concentration by ICP-MS (iCAP Q; Thermo Fisher Scientific, Inc.), according to manufacturer's protocol. Briefly, for ICP-MS, samples were weighed (accurate to 0.1 mg) or HT29 cells were counted prior to microwave digestion using a WX-8000 Microwave Digester (PreeKem Scientific Instruments Co., Ltd.). Following microwave digestion, samples were analyzed by ICP-MS with set parameters for detection.

#### Statistical analysis

Statistical analysis was performed using SPSS version 17.0 (IBM Corp.) and GraphPad Prism 5.0 (GraphPad Software, Inc.). Quantitative variables are presented as the mean ± standard error of the mean. For comparisons of two groups of quantitative data, Student's t-test was used. For multiple comparisons, one-way ANOVA was performed followed by Dunnett's two-tailed test. P<0.05 was considered to indicate a statistically significant difference.

## Results

### 

#### Induction of OCT3 expression by DAC

**Figure 2.**
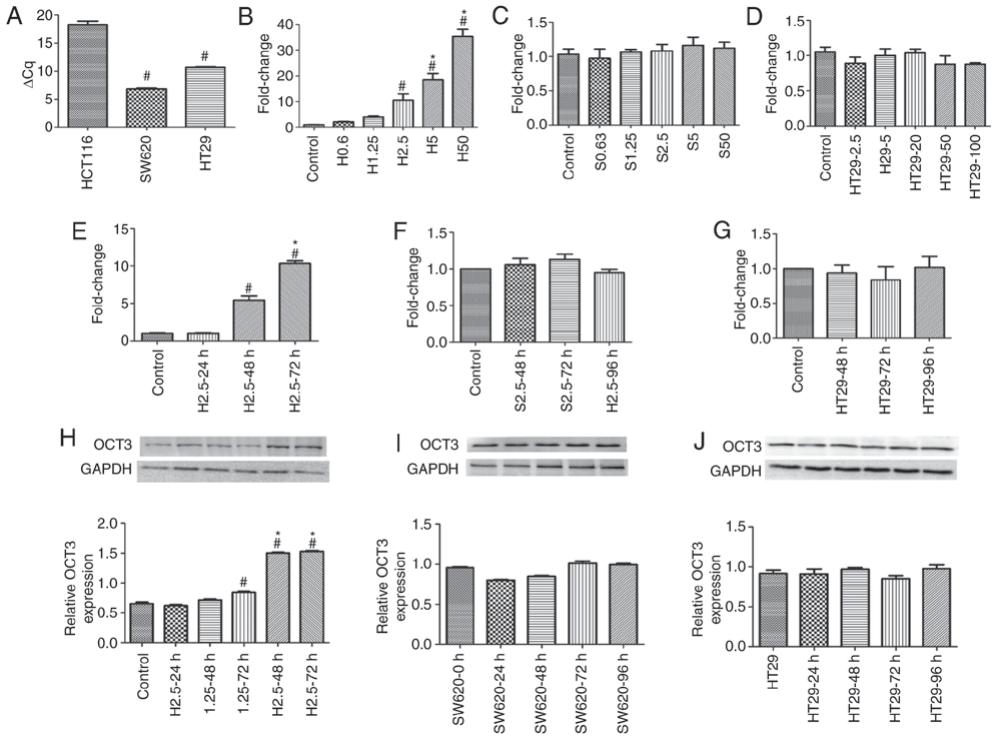
Induction of OCT3 mRNA and protein expression by DAC in three colorectal cancer cell lines. (A) mRNA expression of *SLC22A3* in HCT116, SW620 and HT29 cell. ^#^P<0.05 compared with HCT116 group. (B-D) *SLC22A3* mRNA expression of HCT116 cells, SW620 cells and HT29 cells induced by different concentrations (µM) of DAC, respectively; fold change compared with the control. ^#^P<0.05 compared with Control; *P<0.05 compared with H2.5 group. (E-G) *SLC22A3* mRNA expression induced by treatment with DAC for different durations in the three colon cancer cell lines. ^#^P<0.05 compared with Control; *P<0.05 compared with 48 h DAC group. (H-J) OCT3 protein expression was induced by DAC in a concentration- and time-dependent manner in HCT116 cells, but not in SW620 and HT29 cells. ^#^P<0.05 compared with Control; *P<0.05 compared with 1.25 µM-72 h group. DAC, decitabine; OCT3, organic cation transporter 3.

According to our preliminary experiments, HCT116 and SW620 cells were treated with <50 µM DAC, and HT29 cells were treated with <100 µM DAC, at which cell viability was >80%, in order to detect the effects of DAC on OCT3 expression in the three CRC cell lines. Baseline *SLC22A3* mRNA expression was lowest in HCT116 cells among the three CRC cell lines, and the mRNA expression levels of *SLC22A3* were significantly higher in HT29 and SW620 cells (P<0.05, Fig. 2A). *SLC22A3* mRNA expression was significantly induced in HCT116 cells following treatment with DAC for 72 h in a concentration-dependent manner (Fig. 2B), whereas *SLC22A3* expression was not induced by DAC in SW620 and HT29 cells (Fig. 2C and D). The three CRC cell lines were then treated with 2.5 µM DAC; the results revealed that the induction of *SLC22A3* mRNA expression was time-dependent in HCT116 cells, but not in HT29 or SW620 cells (Fig. 2E-G). The induction of OCT3 protein expression by DAC was similar to that of its mRNA expression. DAC could upregulate OCT3 expression in HCT116 cells, in which the baseline expression of OCT3 was low, in a concentration- and time-dependent manner, but not in SW620 or HT29 cells, in which the baseline expression levels of OCT3 were high (Fig. 2H-J).

#### Methylation status of the SLC22A3 gene promoter region as determined by MSP in CRC cells

**Figure 3.**
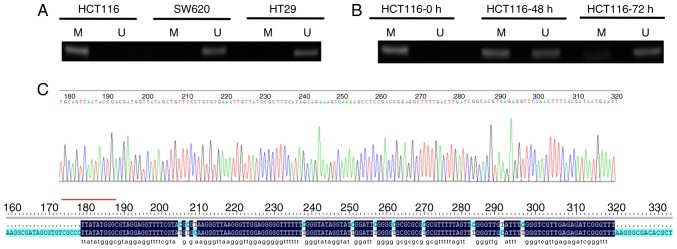
Detection of methylation of the organic cation transporter 3 promoter region in colorectal cancer cells. M, methylated; U, unmethylated.

To investigate the methylation status of the *SLC22A3* gene promoter region in CRC cells, MSP was conducted. Methylated (−) and non-methylated products (+) in SW620 and HT29 cells, and methylated (+) and non-methylated products (−) in HCT116 cells (Fig. 3A). As shown in Fig. 3B, methylation products decreased and non-methylation products increased in HCT116 cells treated with 2.5 µM DAC for 48 and 72 h, respectively. Sequencing revealed that the methylated PCR products were consistent with the target sequence (Fig. 3C). These findings confirmed that the methylation products were the desired products.

#### Influence of DAC on the effects of OXA and OCT3 on CRC cells

**Figure 4.**
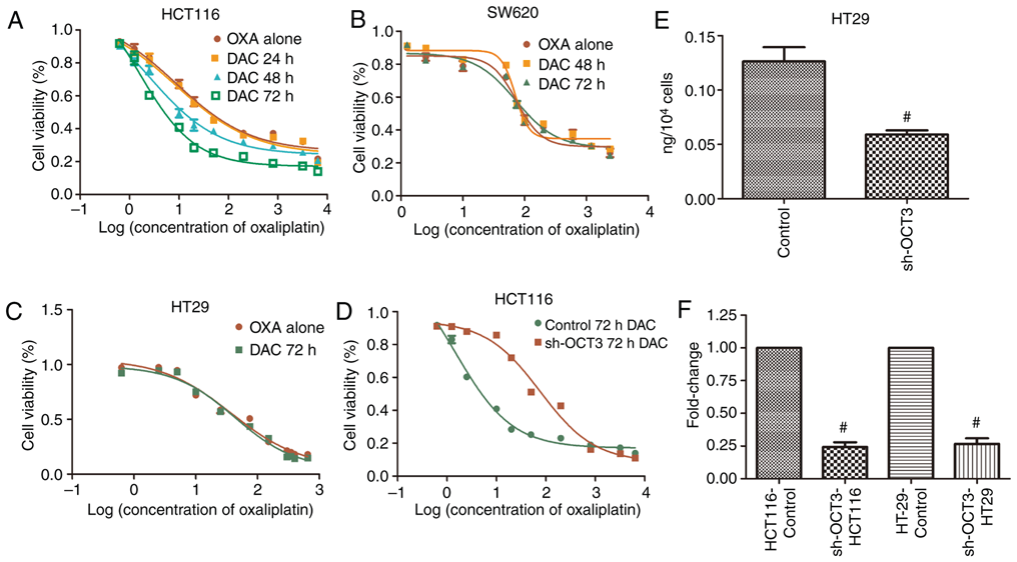
Impact of DAC on the inhibitory effects of OXA and OXA concentrations in colon cancer cells. Alterations in the concentration-activity curves of OXA in colon cancer cells following treatment with 2.5 µM DAC for (A) 0, 24, 48 and 72 h in HCT116 cells, (B) 0, 48 and 72 h in SW620 cells and (C) 0 and 72 h in HT29 cells. Impact of solute carrier family 22 member 3 knockdown (sh-OCT3) on (D) inhibitory effect of OXA on HCT116 cells and (E) OXA concentrations in HT29 cells. (F) Knockdown efficiency on *SLC22A3* in HCT116 and HT29 are both more than 75%. ^#^P<0.05 compared with Control. DAC, decitabine; OCT3, organic cation transporter 3; OXA, oxaliplatin; sh, short hairpin RNA; SLC22A3, solute carrier family 22 member 3.

To explore the influence of DAC on the inhibitory effects of OXA on CRC cells, CRC cell lines were treated with OXA following treatment with 2.5 µM DAC for the indicated durations. The results demonstrated that the concentration-activity curves of OXA declined after HCT116 cells were treated with 2.5 µM DAC for 48 and 72 h, but not for 24 h (Fig. 4A), and the IC_50_ values were also decreased (Table III). There was no significant effect on SW620 cells treated with 2.5 µM DAC for 48 or 72 h, nor HT29 cells for 72 h (Fig. 4B and C). Compared with wild-type HCT116 cells, the concentration-activity curve of OXA in HCT116 cells treated with sh-OCT3 shifted to the right after treatment with DAC (Fig. 4D). The IC_50_ value of OXA was markedly higher in HCT116 cells expressing sh-OCT3 compared with in wild-type HCT116 cells following treatment with DAC (Table III). Furthermore, the effects of OCT3 on the concentration of OXA in HT29 cells were determined; OXA concentration in HT29 cells with *SLC22A3* knockdown (sh-OCT3) was significantly lower than that in the control group (Fig. 4E). The knockdown efficiency on SLC22A3 in HCT116 and HT29 was >75% (Fig. 4F).

#### Direct effect of OCT3 expression on the migration and invasion of CRC cells

**Figure 5.**
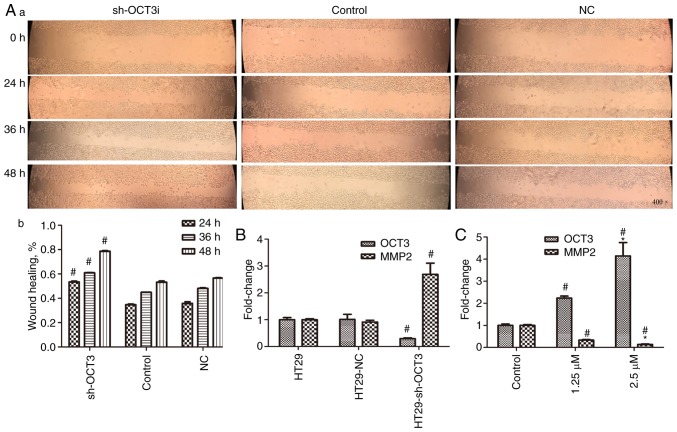
Role of OCT3 expression in cell migration, as determined by wound healing assay, and in cell invasion, as determined by detecting *MMP2* expression. (A-a) Representative images of cell migration (magnification, ×400) and (A-b) wound healing percentage in wild-type and sh-OCT3 HT29 cells at different time points after scratching. (B) Impact of solute carrier family 22 member 3 knockdown (sh-OCT3) on *MMP2* mRNA expression. (C) Impact of treatment with 1.25 and 2.5 µM DAC on *MMP2* mRNA expression. ^#^P<0.05 compared with Control group; *P<0.05 compared with 1.25 µM DAC group. DAC, decitabine; *MMP2*, matrix metalloproteinase 2; NC, negative control; OCT3, organic cation transporter 3; OXA, oxaliplatin; sh, short hairpin RNA.

To explore the role of OCT3 in the migration of CRC cells, wound healing assays were conducted. The results revealed that the percentage of migrating HT29 cells with *SLC22A3* knockdown (sh-OCT3) was significantly higher compared with in the control group (Fig. 5A-a and A-b). To investigate the role of OCT3 in CRC cell invasion, the mRNA expression levels of *MMP2* were detected; *MMP2* is an indicator of cancer cell invasion, whereby elevated *MMP2* expression indicates increased invasion (23,24). The results demonstrated that the expression levels of *MMP2* were significantly higher in HT29 cells with *SLC22A3* knockdown compared with in the control group (Fig. 5B). Consistent with this, *MMP2* mRNA expression in HCT116 cells was reduced in a concentration-dependent manner once OCT3 expression was induced by 1.25 and 2.5 µM DAC (Fig. 5C).

#### Impact of DAC on the effects of OXA on CRC xenografts and the association with OCT3 expression

**Figure 6.**
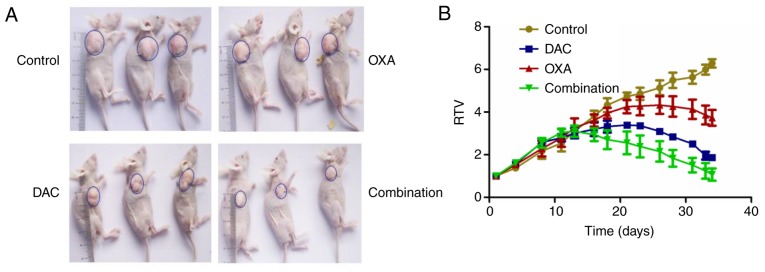
(A) Representative images of xenografts. (B) Changes in the RTV of xenografts in nude mice. DAC, decitabine; OXA, oxaliplatin; RTV, relative tumor volume.

**Figure 7.**
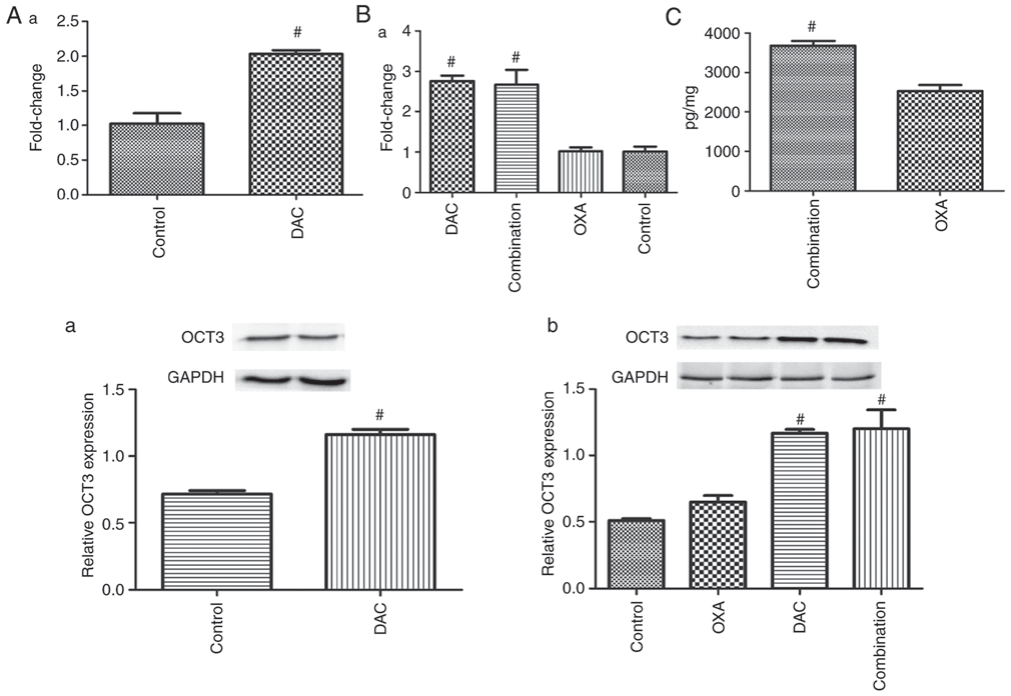
Influence of DAC on the effects of OXA on colon cancer xenografts and the association with OCT3 expression. (A-a) OCT3 mRNA expression in xenografts were detected 7 days after day 1; DAC was administered on days 1, 11 and 21. (A-b) OCT3 protein expression in xenografts after being treated with DAC for 7 days. (B-a) OCT3 mRNA expression in xenografts at the end of the experiment. (B-b) OCT3 protein expression in xenografts at the end of the experiment. ^#^P<0.05 compared with Control group; fold change compared with the control (C) OXA concentration in xenografts from the OXA and combination groups at the end of the experiment. ^#^P<0.05 compared with OXA group. DAC, decitabine; OCT3, organic cation transporter 3; OXA, oxaliplatin.

Representative images of xenografts in nude mice are shown in Fig. 6A. From alterations in RTV curve of xenografts in nude mice, it was revealed that RTV was lowest in the combined DAC and OXA group (Fig. 6B). The inhibitory effect was as follows: Combination group >DAC group >OXA group (Fig. 6A and B). On day 7 following initiation of DAC administration, OCT3 expression in xenografts of mice administered DAC was higher than in xenografts of mice that did not receive DAC (Fig. 7A-a and A-b). At the end of the experiment, OCT3 expression in xenografts of mice administered DAC remained higher than in xenografts of mice that did not receive DAC (Fig. 7B-a and 7B-b). Furthermore, the concentration of OXA in xenografts of mice treated with DAC was higher than in xenografts of mice not treated with DAC (Fig. 7C).

## Discussion

This study demonstrated that the DNA methyltransferase inhibitor DAC could increase the effects of OXA on CRC xenografts, along with an increase in OCT3 expression. DAC upregulated OCT3 expression in CRC cells with OCT3 low expression, which may be of great significance in reversing anticancer drug resistance to OCT3 substrates (25,26), including OXA in CRC. In addition, DAC may inhibit tumor growth by inducing the expression of other tumor suppressor genes (27). Notably, long-term use of DAC may promote resistance, rendering it unable to exert its role in inhibiting DNA methyltransferase 1 (28,29). Tet methylcytosine dioxygenase 2 (*TET2*) encodes a protein that catalyzes the conversion of the modified DNA base methylcytosine to 5-hydroxymethylcytosine, and serves a key role in active DNA demethylation. A recent study (30) suggested that the addition of low-dose vitamin C to DAC may improve complete remission and prolong overall survival compared with DAC alone in elderly patients with AML. Activation of *TET2*, due to the combination of DAC and vitamin C, may be one of the underlying mechanisms. DAC is currently only approved for use in hematological tumors, and clinical trials of DAC in solid tumors are in progress. With the advances made in clinical trials, indications for DAC may expand to solid tumors. The present study provides evidence for the effectiveness of DAC combined with OXA for the treatment of CRC through upregulation of OCT3 expression via DNA demethylation.

Tumor cell migration is one of the critical steps in the invasion and metastasis of malignant tumors. Cell migration assays suggested that OCT3 may inhibit the migration of CRC cells; however, the underlying mechanism requires further investigation. *MMP2* belongs to the MMP family, and its expression is associated with the invasion and metastasis of CRC (31,32). Therefore, *MMP2* was selected as a marker to indicate the possible role of OCT3 in the biological behavior of malignant tumors by detecting the impact of OCT3 expression on *MMP2* expression. The results revealed that *MMP2* expression was upregulated in *SLC22A3-*knockdown CRC cells, which indicated that OCT3 may inhibit the invasion and metastasis of CRC by reducing *MMP2* expression. However, the mechanism by which OCT3 inhibits *MMP2*, directly or indirectly, is unclear. Consistent with the present results, Fu *et al* (12) recently revealed that the expression of *SLC22A3* in non-tumorous tissues of patients with familial esophageal cancer is significantly downregulated, and adenosine-to-inosine RNA editing of this gene leads to downregulation of its expression and is significantly associated with lymph node metastasis. Further investigations have reported that OCT3 can directly bind α-actinin-4 (*ACTN4*), and may inhibit *ACTN4*-mediated actin cross-linking and cell migration (12). A limitation of the present study is that, for migration assays, 1% serum-containing medium was added to the culture dish following wound generation, instead of serum-free medium. Although 1% serum-containing medium has little effect on cell proliferation, it may not completely exclude the effect of cell proliferation on the experiment.

In conclusion, OCT3 may directly inhibit the malignant biological behavior of cancer and may be considered a novel target for intervention in CRC. OCT3-specific inhibitors have recently been reported (33,34), and will be useful tools for further investigating the function of OCT3.

## Figures and Tables

**Table I.** Primers for reverse transcription-quantitative polymerase chain reaction analysis.

Oligonucleotide name	Source	Sequence (5′-3′)	Amplified fragment length (bp)
*SLC22A3*-F	Human	CCCTGGAATTGCCTACTTCA	102
*SLC22A3*-R	Human	GACTCAGGGACCACCCAGTA	
β-actin-F	Human	CATCGAGCACGGCATCGTCA	211
β-actin-R	Human	TAGCACAGCCTGGATAGCAAC	
*MMP2*-F	Human	GATACCCCTTTGACGGTAAGGA	112
*MMP2*-R	Human	CCTTCTCCCAAGGTCCATAGC	

F, forward; R, reverse; *MMP2*, matrix metalloproteinase 2; *SLC22A3*, solute carrier family 22 member 3.

**Table II.** Primers for methylation-specific polymerase chain reaction analysis.

Oligonucleotide name	Source	Sequence (5′-3′)
*SLC22A3* M-F	Human	TATATGGGCGTAGGAGGTTTC
*SLC22A3* M-R	Human	AAACCCGATCTCTCAACGAC
*SLC22A3* U-F	Human	TTTTATATGGGTGTAGGAGGTTTTT
*SLC22A3* U-R	Human	ACTTCTAAAACCCAATCTCTCAACA

F, forward; R, reverse; M, methylated; U, unmethylated; *SLC22A3*, solute carrier family 22 member 3.

**Table III.** Effects of DAC on IC_50_ of OXA in colorectal cancer cells.

	2.5 µM DAC			
	
Cell line	0 h	24 h	48 h	72 h
HCT116	8.64 (3.68–20.29)	8.37 (3.11–22.47)	2.38 (0.70–8.1)	1.50 (0.62–3.62)
SW620	71.94 (56.92–90.91)		70.22 (63.78–77.33)	74.41 (64.1–86.37)
HT29	37.58 (23.76–59.43)			42.89 (31.10–59.16)
HCT116-sh-OCT3				76.61 (55.59–105.6)

Data are presented as the mean (95% CI). sh, short hairpin RNA; OCT3, organic cation transporter 3.

## Data Availability

All data generated or analyzed during this study are included in this published article.

## Abbreviations

OCT3: organic cation transporter 3
*SLC22A3*: solute carrier family 22 member 3
DAC: decitabine
OXA: oxaliplatin
MMP2: matrix metalloproteinase-2
RT-qPCR: reverse transcription-quantitative polymerase chain reaction
ICP-MS: inductively coupled plasma mass spectrometry
FBS: fetal bovine serum
EMT: epithelial-mesenchymal transition
MSP: methylation-specific polymerase chain reaction
CRC: colorectal cancer
